# Excited States by Coupling Piris Natural Orbital Functionals with the Extended Random-Phase Approximation

**DOI:** 10.1021/acs.jctc.3c01194

**Published:** 2024-02-14

**Authors:** Juan Felipe Huan Lew-Yee, Iván Alejandro Bonfil-Rivera, Mario Piris, Jorge M. del Campo

**Affiliations:** † Departamento de Física y Química Teórica, Facultad de Química, Universidad Nacional Autónoma de México, México City C.P. 04510, Mexico; ‡ Donostia International Physics Center (DIPC), 20018 Donostia, Spain; § Kimika Fakultatea, Euskal Herriko Unibertsitatea (UPV/EHU), 20018 Donostia, Spain; ∥ IKERBASQUE, Basque Foundation for Science, 48013 Bilbao, Spain

## Abstract

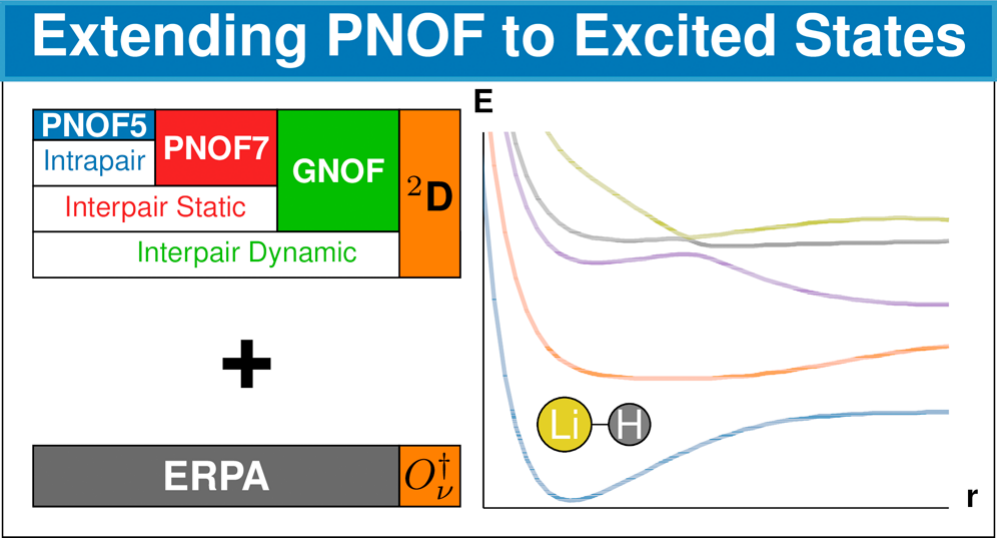

In this work, we explore the use of Piris natural orbital functionals (PNOFs) to calculate excited-state energies by coupling their reconstructed second-order reduced density matrix with the extended random-phase approximation (ERPA). We have named the general method PNOF-ERPA, and specific approaches are referred to as PNOF-ERPA0, PNOF-ERPA1, and PNOF-ERPA2, according to the way the excitation operator is built. The implementation has been tested in the first excited states of H_2_, HeH^+^, LiH, Li_2_, and N_2_ showing good results compared to the configuration interaction (CI) method. As expected, an increase in accuracy is observed on going from ERPA0 to ERPA1 and ERPA2. We also studied the effect of electron correlation included by PNOF5, PNOF7, and the recently proposed global NOF (GNOF) on the predicted excited states. PNOF5 appears to be good and may even provide better results in very small systems, but including more electron correlation becomes important as the system size increases, where GNOF achieves better results. Overall, the extension of PNOF to excited states has been successful, making it a promising method for further applications.

## 1 Introduction

Excited states^1,2^ are important for the description of photochemical^3^ and electrochemical processes,^4^ fluorescence and phosphorescence phenomena,^5^ spectroscopy,^6^ and chemical reaction mechanisms,^7,8^ with a variety of cutting-edge chemical applications such as the development of new materials for organic solar cells^9^ and batteries.^10−12^ The energy of these states can be calculated using the configuration interaction (CI) method; however, this becomes too expensive even for low levels of CI, although some variations have been developed to address this issue.^13^ There are other electronic structure tools that allow studying excited species at a more affordable cost, such as the equation-of-motion coupled-cluster (EOM-CC)^14−16^ approach, the time-dependent density functional theory (TD-DFT),^17^ and the random-phase approximation,^18^ but the accuracy achieved is not as good as desired and the picture becomes more complex when it comes to static correlation, since multireference methods are required.^19,20^

In this context, the one-particle reduced density matrix (1RDM) functional theory (1RDMFT)^21,22^ appears to be a suitable approach to study excited states taking into account electronic correlation effects, including the strong ones. In particular, time-dependent 1RDMFT in its adiabatic linear response formulation has been developed^23,24^ to calculate the energies of excited states and oscillator strengths;^25^ however, a solid foundation for a dynamic 1RDMFT is still an open challenge.^26^ On the other hand, an ensemble version of 1RDMFT has recently been proposed^27^ to calculate the energies of selected low-lying excited states, although it will require more efficient numerical minimization schemes for its future success.^28^ In this article, we shall use the extended random-phase approximation^29,30^ within the 1RDMFT framework in the natural orbital representation. Specifically, we will employ the Chatterjee and Pernal’s formulation^31^ that relies on the 1RDM and the two-particle reduced density matrix (2RDM) of the ground state. The method can be elegantly derived from the formally exact Rowe’s excitation operator equation-of-motion,^32^ and has been used^33−35^ successfully with the RDMs corresponding to the wave function of the antisymmetrized product of strongly orthogonal geminals (APSG).

In this vein, it has been shown^36,37^ that the APSG approach is equivalent to the Piris natural orbital functional 5 (PNOF5),^38^ except for a phase factor. PNOFs^39,40^ are based on the reconstruction of the 2RDM constrained to certain bounds due to the N-representability conditions,^41^ and belong to the JKL-only family of natural orbital functional (NOFs), where J and K refer to the usual Coulomb and exchange integrals, while L denotes the exchange-time-inversion integral.^42^ The latter is relevant for excited states due to the fact that it allows the time-evolution of the occupation numbers, contrary to the stationarity of the occupation numbers demonstrated^23,43^ for the JK-only NOFs.

The performance of PNOFs has achieved chemical accuracy in many cases,^44^ with electron-pairing-based functionals^45^ being particularly successful in describing nondynamic electron correlation, namely PNOF5,^37^ PNOF6,^46^ and PNOF7.^47^ Furthermore, the most recent functional, GNOF^48^ has extended the success of PNOF to a balanced electron correlation regime,^49^ as has been observed in the study of a variety of chemical systems such as hydrogen models in one, two, and three dimensions,^50^ iron porphyrin multiplicity,^51^ carbenes singlet–triplet gaps,^52^ and all-metal aromaticity.^53^ Motivated by this success, the extension of PNOF to excited states becomes tempting, which can be accomplished by introducing their approximate RDMs into the ERPA equations. The PNOF-ERPA approach has the potential to be a viable substitute for multireference wave function techniques in the modeling of excited states.

This account is organized as follows. In Section 2, we briefly review the equations of the ERPA and the PNOFs used. Next, we give some computational details of the calculations in Section 3. In Section 4, the performance of these approaches is tested in detail on potential energy curves (PECs) of diatomic molecules with an increasing number of electrons. Finally, conclusions are listed in Section 5.

## 2 Theory

In this section, we summarize the ERPA equations as used in this work to couple them with the reconstructed 2RDM of PNOFs in terms of occupation numbers. A detailed description of ERPA can be found in the work of Chatterjee and Pernal.^31^ We address only singlet states, so we adopt a spin-restricted formalism in which a single set of orbitals is used for the alpha and beta spins.

### 2.1 ERPA

In the context of the equation-of-motion method, the expectation value of the double commutator developed by Rowe^32^ for a system described by a Hamiltonian *Ĥ* is defined as
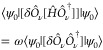
1where ω corresponds to the excitation energy,  is the excitation operator that applied to the ground state |ψ_0_⟩ produces the excited state |ψ_ν_⟩, namely
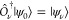
2whereas  de-excitates from |ψ_ν_⟩ to |ψ_0_⟩, and satisfies the consistency condition to ensure the orthogonality of the ground and excited states, that is
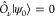
3The equations to solve are obtained by using an excitation operator, with its simplest form including only single nondiagonal excitations, which we have called ERPA0. Therefore,  is approximated as
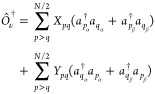
4where *X*_*pq*_ and *Y*_*pq*_ are coefficients to be determined. In the following, the indices *p*, *q*, *r*, *s*, *t*, *u*, and *v* will be used for spatial orbitals, and α and β for spin.

Taking the variation of the adjoint of the excitation operator and substituting in eq 1, it is obtained
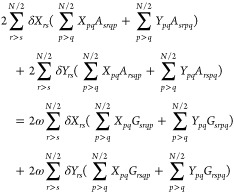
5with
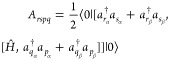
6and
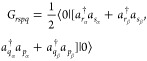
7where a factor of “2” has been added to eq 5 and compensated with a factor of “1/2” in eqs 6 and 7 for convenience.

Considering the 1RDM in its diagonal representation
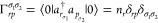
8the 2RDM
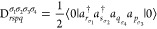
9and recalling the restricted-spin formalism (*n*_*p*_ = *n*_*p*_^α^ = *n*_*p*_^β^, ϕ_*p*_ = ϕ_*p*_^α^ = ϕ_*p*_^β^, and consequently *D*^αααα^ = *D*^ββββ^, *D*^αβαβ^ = *D*^βαβα^), the elements of *A*_*rspq*_ can be expressed as
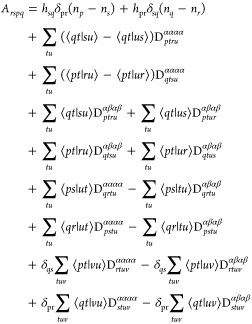
10where *h*_*pq*_ represent the elements of the core Hamiltonian matrix, and ⟨*pq*|*rs*⟩ corresponds to the electron repulsion integrals in the basis of spatial natural orbitals. Applying the commutator and considering the 1RDM in its diagonal representation of natural orbitals and occupation numbers, the elements of *G*_*rspq*_ are given by
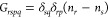
11Grouping the terms of eq 5 by the variations of δ*X*_*rs*_ and δ*Y*_*rs*_ leads to two type of equations. Furthermore, these can be simplified by considering the Kronecker deltas of eq 11 and the conditions *r* > *s* and *p* > *q* imposed by the sums to give
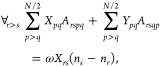
12
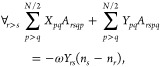
13where we have used the fact that
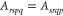
14These equations can be cast in matrix form to the generalized eigenvalue problem given by
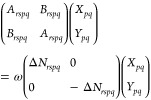
15where we have used
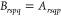
16
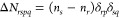
17Equation 15 can be written only in terms of **A**, but these auxiliary variables allow identifying the appropriate blocks to reformulate the problem in a more compact form, as
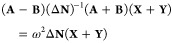
18
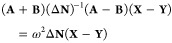
19which resemble what is done to reduce the generalized eigenvalue problem of TD-SCF.^54^

Recalling the fact that when having occupation numbers of exactly ones and zeros, the PNOFs ground state goes to the Hartree–Fock limit, it is also interesting that in this limit PNOF-ERPA0 becomes equivalent to the TD-HF method. This can be seen from eq 17, where having only ones and zeros as occupation numbers makes Δ*N* the identity matrix with some additional zeros that can be discarded. Hence, eq 15 introduces electron correlation to excited states through the occupation numbers.

We can go beyond by including single-diagonal excitations in the operator, a procedure that we labeled as ERPA1. For this case, the excitation operator is defined as
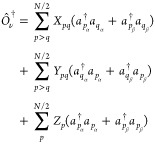
20where *X*_*pq*_, *Y*_*pq*_, and *Z*_*p*_ are the coefficients to be determined. Substituting in eq 1 and following a similar procedure than before, we arrive to
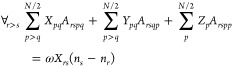
21
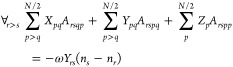
22
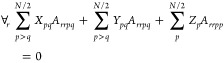
23

Defining the auxiliary variables
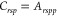
24
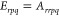
25
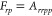
26these equations can be cast in matrix form as
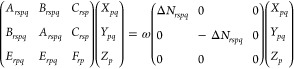
27Furthermore, the problem can be reformulated in a more compact form as
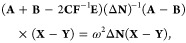
28
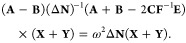
29

Unfortunately, both ERPA0 and ERPA1 violate the consistency condition, and hence, the excitation energies deteriorate. This condition may be enforced for two-electron systems by including double diagonal excitations, namely, ERPA2,^31^ with the excitation operator given by
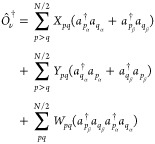
30Substituting in eq 1, imposing the consistency condition and taking into account that for two-electron systems the RDMs of order higher than two vanish, the equations obtained for two-electron systems are extended to any N-electron spin-compensated system, resulting in the following generalized eigenvalue problem
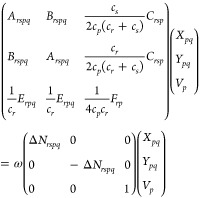
31where
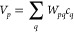
32and *c*_*q*_ is the square root of the occupation number *n*_*q*_, that is, . An important difference between the APSG-ERPA and PNOF-ERPA approaches is the square root sign that is determined in the optimization process of APSG, but it is chosen to reproduce the functional form of PNOF, as detailed in the next section.

### 2.2 PNOF

Having the equations of ERPA0, ERPA1, and ERPA2, we only need to express the elements of matrix **A** given in eq 10, according to the 2RDM reconstructions of the PNOFs that we consider in this work. This allowed us to implement the PNOF-ERPA0, PNOF-ERPA1 and PNOF-ERPA2 approximations. Next, we describe the 2RDMs corresponding to PNOF5,^37^ PNOF7,^47^ and GNOF.^48^

**Figure 1:**
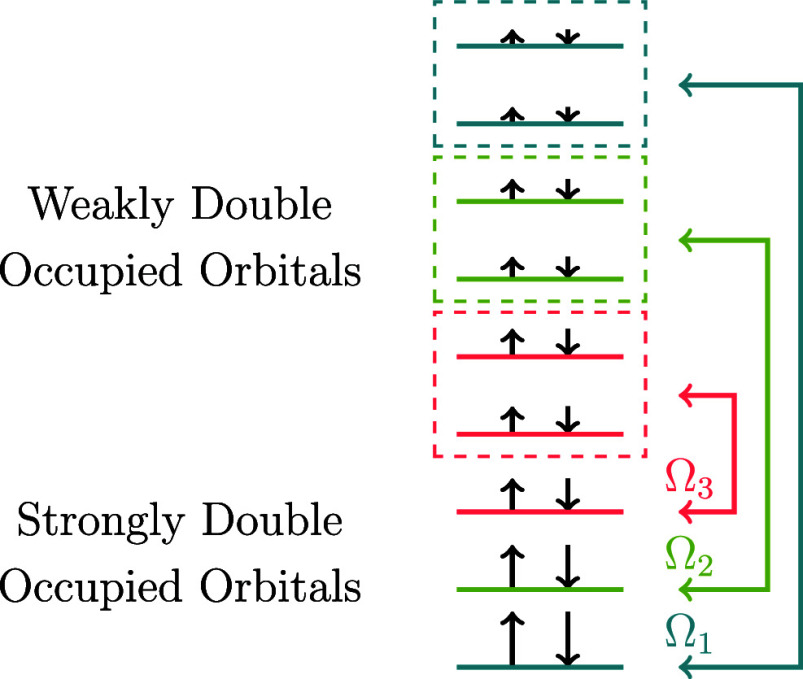
Example of the pairing scheme used in PNOF for a singlet state of a system with 6 electrons (*N* = 6). There are *N*/2 = 3 subspaces, namely, Ω_1_, Ω_2_, and Ω_3_. In this example, an extended pairing scheme with *N*_*g*_ = 2 have been used, therefore there are two weakly double-occupied natural orbitals coupled to each strongly double-occupied natural orbital.

The aforementioned functionals use an electron-pairing scheme, as depicted in Figure 1. Given a system with *N* electrons in the orbital space Ω, we divide the latter into *N*/2 mutually disjoint subspaces Ω_*g*_, so each orbital belongs only to one subspace. A given subspace Ω_*g*_ contains one strongly double-occupied orbital ϕ_*g*_ below the level *N*/2, and *N*_*g*_ weakly double-occupied orbitals above it, and its occupation numbers sum to “1”. The case where *N*_*g*_ = 1 is called the orbital perfect-pairing scheme, while *N*_*g*_ > 1 is called the extended-pairing scheme. It is important to note that orbitals satisfying the pairing conditions are not required to remain fixed throughout the orbital optimization process. The 2RDM (**D**) is divided into intra- and intersubspace contributions, corresponding to the intrapair electronic correlation, that is, the contribution of the orbitals in a given subspace Ω_*g*_, and the interpair electronic correlation, that is, the contribution between the orbitals of a subspace Ω_*g*_ with those of a different subspace Ω_*f*_, *f* ≠ *g*.

The simplest way to meet all N-representability constraints imposed^39^ on the 2RDM of PNOF leads to the independent pairs model PNOF5, where only intrapair (intrasubspace) electron correlation is taken into account, namely
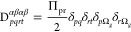
33where Kronecker deltas have a standard meaning, for example  is one if the natural orbital ϕ_*r*_ belongs to the subspace Ω_*g*_, and zero otherwise. The matrix elements are defined as Π_*pr*_ = *c*_*p*_*c*_*r*_, where *c*_*p*_ is defined by the square root of the occupation numbers according to the rule
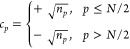
34that is, the phase factor of *c*_*p*_ is chosen to be +1 for the strongly occupied orbital of a given subspace Ω_*g*_, and −1 otherwise. On the other hand, the intersubspace contributions (Ω_*g*_ ≠ Ω_*f*_) are assumed Hartree–Fock-like
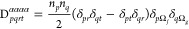
35
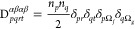
36

To go beyond the independent-pair approximation, the electron correlation between pairs (subspaces) is introduced. In all post-PNOF5 reconstructions, the parallel spin blocks have remained Hartree–Fock-like as in eq 35, while the opposite spin contribution between pairs (subspaces) is different.

For PNOF7, it was introduced the function
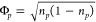
37so the interpair opposite spin contribution is given by
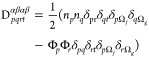
38

Note that Φ_*p*_ has significant values only when the occupancies differ substantially from “1” and “0”. Consequently, PNOF7 can recover the static correlation between pairs, but it lacks interpair dynamic electron correlation.

GNOF introduces the concept of dynamic occupation numbers as
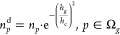
39with the hole given by *h*_*g*_ = 1 – *n*_*g*_ and . The interpair opposite spin contribution is then given by
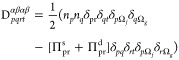
40with
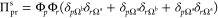
41
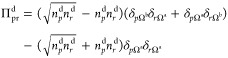
42where Ω^b^ denotes the subspace composed of orbitals below the level *N*/2 (*p* ≤ *N*/2), while Ω^a^ denotes the subspace composed of orbitals above the level *N*/2 (*p* > *N*/2). Observe that the interactions between orbitals belonging to Ω^b^ are not considered in the Π matrices of GNOF.

The matrix **Π**^d^ accounts for the dynamic correlation between subspaces in accordance with Pulay’s criterion, which establishes an occupancy deviation of approximately 0.01 with respect to “1” or “0” for a natural orbital to contribute to the dynamic correlation, while larger deviations contribute to nondynamic correlation. **Π**^s^ from the PNOF7 functional form is conserved.

## 3 Computational Details

The NOF calculations have been carried out using an extended pairing approach, that is, we correlate all electrons into all available orbitals for a given basis set, which today is not possible for large systems with current wave function-based methods. The number of weakly double-occupied orbitals coupled to each strongly double-occupied orbital is indicated as *N*_cwo_ below the plot of each system. CI and TD-DFT calculations were carried out for comparison using the Psi4^55,56^ software. All calculations have been performed using a def2-TZVPD basis set,^57,58^ except for the N_2_ calculation that was performed using a cc-pVDZ basis set.^59^

The equations of PNOF coupled to ERPA0, ERPA1, and ERPA2 have been implemented in the DoNOF^60^ and in PyNOF software.^61^ It is important to notice that several techniques have been developed to avoid explicit storage of **A** and **B** matrices, as well as the full diagonalization of large matrices.^62,63^ In particular, the algorithm of Stratmann, Scuseria, and Frisch,^54^ that take advantage of the fact that the excitation energies appear in pairs, may be applicable to ERPA0 and ERPA1, although with some modifications, as the vectors **X** + **Y** and **X** – **Y** are not orthonormal as in TD-SCF, but instead the orthonormality is hold by the vectors **X** – **Y** and **ΔN**(**X** + **Y**). This can be seen for ERPA0 by rewriting the reduced equations as
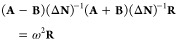
43
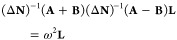
44where **R** = **ΔN**(**X** + **Y**) and **L** = (**X** – **Y**) are the right and left eigenvectors of the (**A** – **B**)(**ΔN**)^−1^(**A** + **B**)(**ΔN**)^−1^ matrix. Using this approach would allow us to iteratively compute a selected number of excitation by diagonalizing small matrices. A similar approach can be applied to ERPA1. However, some details of the algorithm must be explored, for example, the possibility of Δ**N** being not invertible, as well as the symmetry of the matrices involved in ERPA1. Furthermore, this approach may not be applicable to ERPA2, as the paired structure of the eigenvalues is lost in this case. For the purpose of this work, we are solving ERPA0 by eq 18, ERPA1 by eq 28 and ERPA2 by eq 31 by performing full diagonalization of the involved matrices.

## 4 Results and Discussion

In this section, we present the ground and excited state PECs of model systems, namely, H_2_, HeH^+^, LiH, and Li_2_, computed with PNOFs coupled to ERPA0, ERPA1, and ERPA2. These molecules are of interest due to their low number of electrons that allow the results to be compared directly with the values of the FCI method. In addition, we also present the PEC of N_2_, a larger system that involves breaking a triple bond.

### 4.1 ERPA0 vs ERPA1 vs ERPA2: H_2_, HeH^+^, and LiH

**Figure 2:**
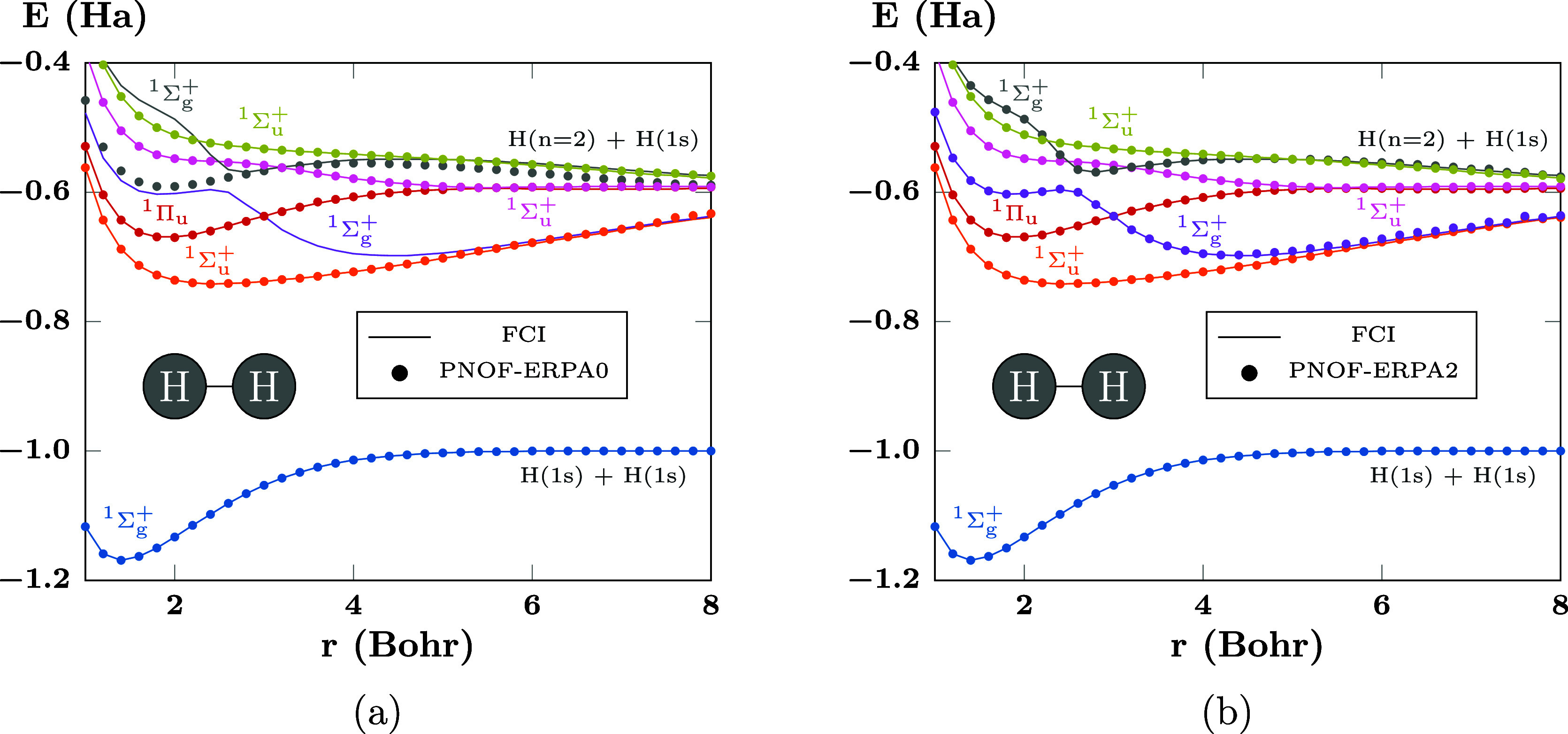
PECs of the first states of H_2_ computed using (a) PNOF-ERPA0 and (b) PNOF-ERPA2. FCI results are shown as solid lines as a reference. There are *N*_cwo_ = 17 orbitals paired to each strongly double-occupied orbital. The first curve corresponds to the ground state.

The cases of H_2_ and HeH^+^ are remarkable since only intrapair (and no interpair) contributions to the electron correlation are required. In these cases, PNOF5, PNOF7, and GNOF converge to the same functional form. We start with the simplest of these molecules, the homonuclear diatomic H_2_. The energies of the ground and excited states are presented in Figure 2, with the PNOF results as circle marks and the FCI reference values as solid lines. In particular, Figure 2a,b presents the results of PNOF-ERPA0 and PNOF-ERPA2, respectively.

From Figure 2a, it can be seen that the ground state (^1^Σ_*g*_^+^, blue) and several excited states (^1^Σ_*u*_^+^ orange, ^1^Π_*u*_ red, ^1^Σ_*u*_^+^ pink, ^1^Σ_*u*_^+^ golden) PECs are in good agreement with FCI. On the other hand, excited states such as those of ^1^Σ_*g*_^+^ gray and purple marks agree well with FCI in some but not all the domain, this is caused by a lost state that makes it impossible to capture the avoided crossing between the gray and purple curves around 2.6 Bohr. These deviations can be tracked to a violation of the consistency condition. In this regard, PNOF-ERPA1, which includes single diagonal excitations, provides almost the same results as PNOF-ERPA0, and the problem can only be solved by including diagonal double excitations.^31^ The results of PNOF-ERPA2 presented in Figure 1b show that it can recover the lost state; consequently, the avoided crossing and the shapes of the gray and purple curves are well described. It should be noted that, even though PNOF-ERPA0 loses the avoided crossing, it is still able to describe the intersection between the pink and gray curves at 3.2 Bohr.

**Figure 3:**
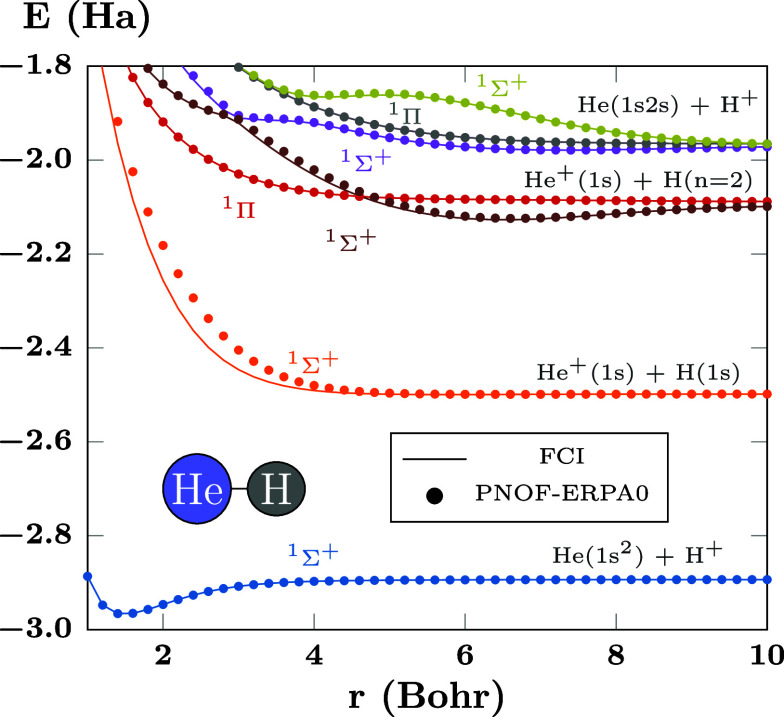
PECs of the first states of HeH^+^ computed using PNOF-ERPA0 and FCI. There are *N*_cwo_ = 17 orbitals paired to each strongly double-occupied orbital. The first curve corresponds to the ground state.

**Figure 4:**
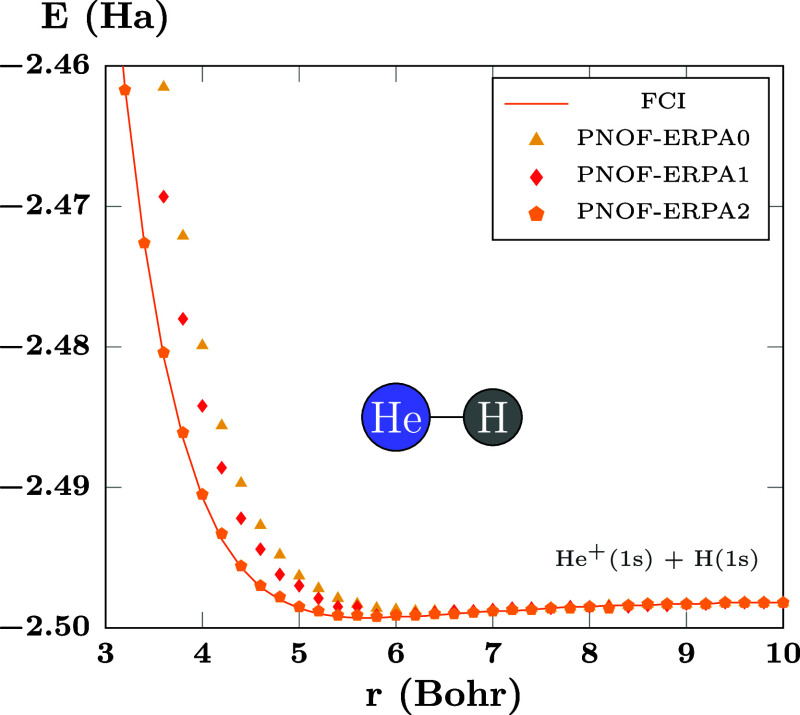
First excited state of HeH^+^. There are *N*_cwo_ = 17 orbitals paired to each strongly double-occupied orbital.

On the other hand, Figure 3 shows the PECs of HeH^+^, a diatomic heteronuclear charged system computed with PNOF-ERPA0. It can be seen that most of the results accurately reproduce the FCI values, including the avoided crossing between the ^1^Σ^+^ brown and purple curves at 3.0 Bohr, and the crossing between the ^1^Σ^+^ brown and ^1^Π red curves at 4.6 Bohr. In this case, no state has been lost, although the ^1^Σ^+^ orange curve that corresponds to the first excitation exhibits some deviations at a distance separation below 5 Bohr. This can be improved, as can be seen in Figure 4, where going from ERPA0 (triangles) to ERPA1 (diamonds) provides better results and going to ERPA2 (pentagons) makes the values accurate. It is worth noting that no significant differences are observed beyond 6 Bohr of bond length.

**Figure 5:**
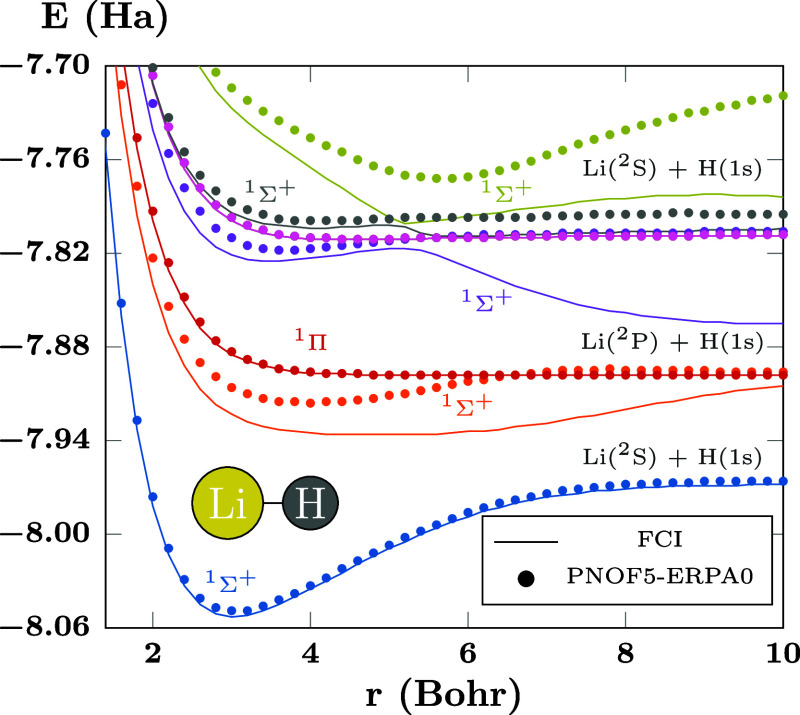
PECs of the excited states of LiH computed using PNOF5-ERPA0 and FCI. There are *N*_cwo_ = 12 orbitals paired to each strongly double-occupied orbital. The first curve corresponds to the ground state.

The lithium hydride, with two electron pairs, represents a more correlated system, being the first system in this work to present interpair electron correlation. The energies of the first states of LiH calculated with PNOF5-ERPA0 are presented in Figure 5, where it can be seen that the method is capable of capture the general profile of the PECs. However, there are some details worth discussing, especially since this system has been used as a model due to its well-known avoided crossings.^64^

The ^1^Σ^+^ orange curve tends to increase in energy too soon as the molecule is dissociated, and the main deviation occurs around 7 Bohr, where a strongly avoided crossing between the ^1^Σ^+^ orange and blue curves occurs. Similarly, PNOF5-ERPA0 does not describe well the avoided crossing between the ^1^Σ^+^ orange and purple curves at 10 Bohr, and the avoided crossing between the ^1^Σ^+^ purple and gray curves at around 5.4 Bohr. The curves involved in these avoided crossings are affected by their energy predictions. Moving from ERPA0 to ERPA1 and ERPA2 does not significantly affect the points that are already accurate in Figure 5, that is, those of the blue, red, and pink curves, and in any case improves slightly their accuracy; then, in the following, we will remove these curves and focus on the other PECs for clarity.

**Figure 6:**
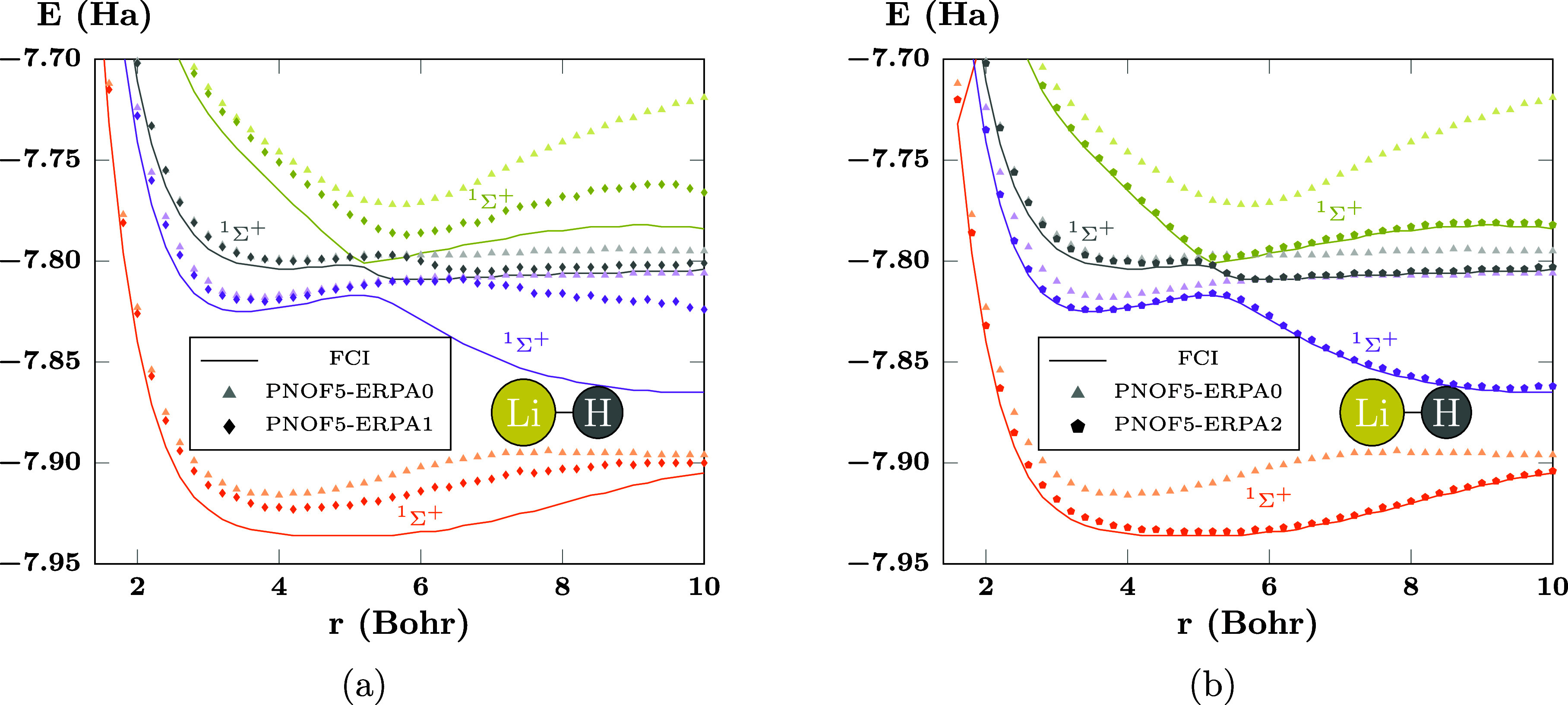
PECs of selected states of LiH computed using (a) PNOF5-ERPA1 and (b) PNOF5-ERPA2. FCI results are presented as solid lines, and PNOF5-ERPA0 values are shown as attenuated triangles on the background for comparison. There are *N*_cwo_ = 12 orbitals paired to each strongly double-occupied orbital. The ground state is not shown.

Figure 6a presents the improved curves achieved by PNOF5-ERPA1 (diamonds), and the values of PNOF5-ERPA0 (triangles) are shown attenuated on the background as a reference. It can be seen that ERPA1 improves the ERPA0 results by allowing the ^1^Σ^+^ gray curve to become closer to the FCI reference. The ^1^Σ^+^ orange, golden, and gray curves are qualitatively improved by taking the appropriate shape. Although PNOF5-ERPA1 is not completely accurate, it shows that in this case the single diagonal excitations may become significant to go beyond the PNOF5-ERPA0 approximation. Furthermore, the PNOF5-ERPA2 approach is capable of recovering all avoided crossings in the studied region, as shown in Figure 6b, with marks that are very close to the FCI results. It is clear that going from ERPA0 to ERPA1 and ERPA2 improves the results, as the marks become closer to the lines of the FCI reference.

**Figure 7:**
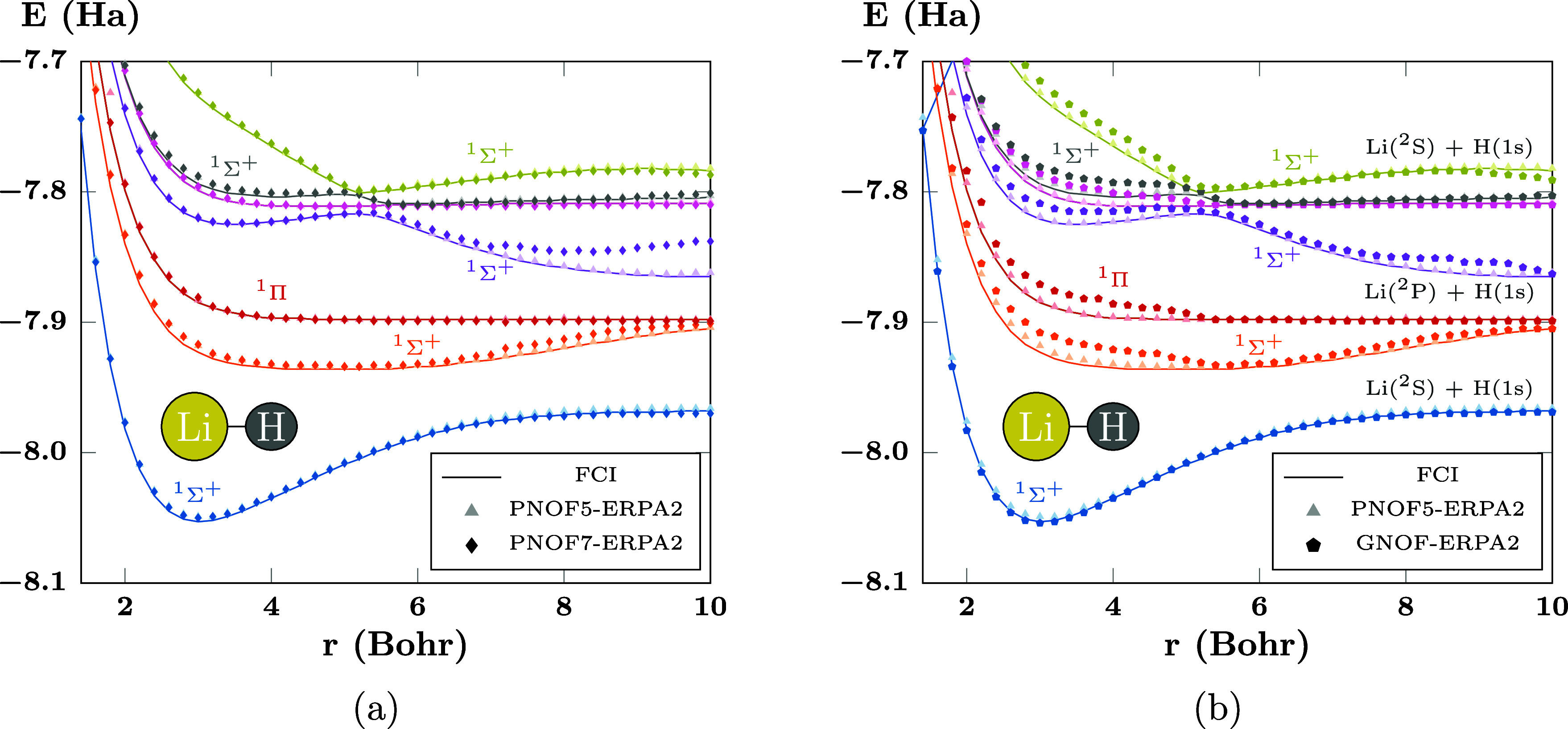
PECs of the first states of LiH computed using (a) PNOF7-ERPA2 and (b) GNOF-ERPA2. There are *N*_cwo_ = 12 orbitals paired to each strongly double-occupied orbital. The ground state is shown in blue.

**Figure 8:**
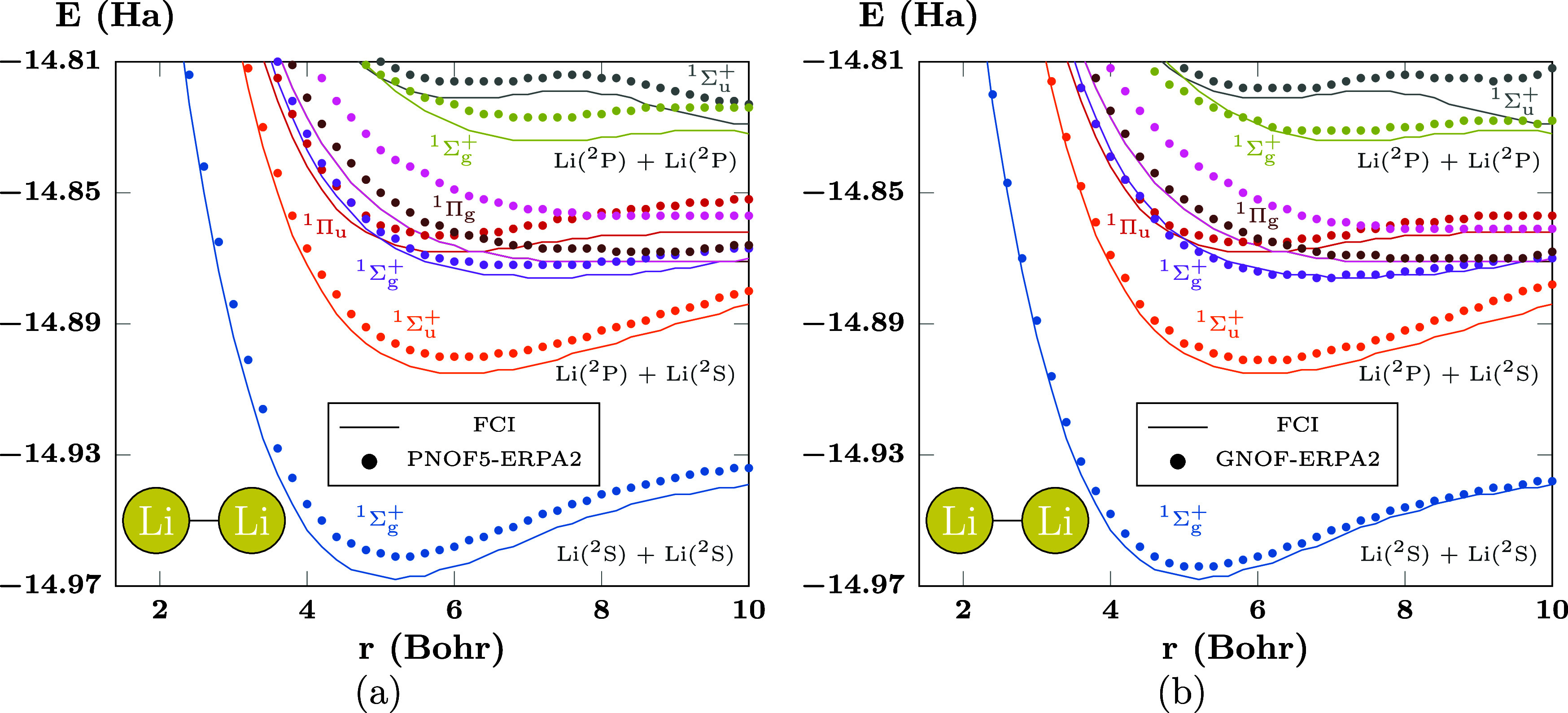
PECs of the first states of Li_2_ computed using (a) PNOF5-ERPA2 and (b) GNOF-ERPA2. There are *N*_cwo_ = 10 orbitals paired to each strongly double-occupied orbital. The first curve corresponds to the ground state.

### 4.2 PNOF5 vs PNOF7 vs GNOF: LiH and Li_2_

Since LiH has interpair electron correlation, PNOF5, PNOF7, and GNOF provide different results, as previously reported for the ground state,^49^ with PNOF5 presenting the highest energies, GNOF being close to FCI, and PNOF7 remaining at intermediate energies; although the energy differences are small. Regarding the excited states, as for this system, the dynamic correlation is dominant around the binding region; the PNOF7-ERPA2 PECs show no significant differences with those of PNOF5-ERPA2, but present some deviations in the dissociation region beyond 7.0 Bohr, as can be seen in Figures 7a. On the other hand, the GNOF-ERPA2 picture also resembles that of PNOF5-ERPA2 but with some small deviations near the binding region, as can be seen in Figures 7b. This is most evident for the ^1^Σ^+^ orange and ^1^Π red curves around 4 Bohr of interatomic distances. The pointed discrepancies could be related to the fact that PNOF5 is strictly N-representable, while PNOF7 and GNOF satisfy only some necessary N-representability conditions. Due to the low number of electrons in LiH, the violations of the N-representability appear to have a more significant contribution over the consideration of the interpair electron correlation. However, this relation changes as the number of electrons increases, as is seen in the next system.

The effect of the interpair electron correlation becomes more evident in Li_2_, with three electron pairs. As the behavior of ERPA0, ERPA1 and ERPA2 has been established, here we use ERPA2 directly and look for the difference between PNOFs. The PECs of Li_2_ computed with PNOF5-ERPA2 are presented in Figure 8a, where it can be seen that PNOF5-ERPA2 achieves qualitatively good results. However, although the curves have been recovered, further inspection shows deviations in almost all cases relative to the FCI lines. Going beyond the independent-pair model becomes important, as can be seen in Figure 8b where the excited state energies computed with GNOF-ERPA2 are presented. In this case, all the marks become closer to the FCI curves, especially those corresponding to the blue, purple, and golden ^1^Σ_g_^+^ curves, as well as the gray ^1^Σ_u_^+^ PECs, thus providing more accurate values due to the better treatment of the electron correlation.

**Figure 9:**
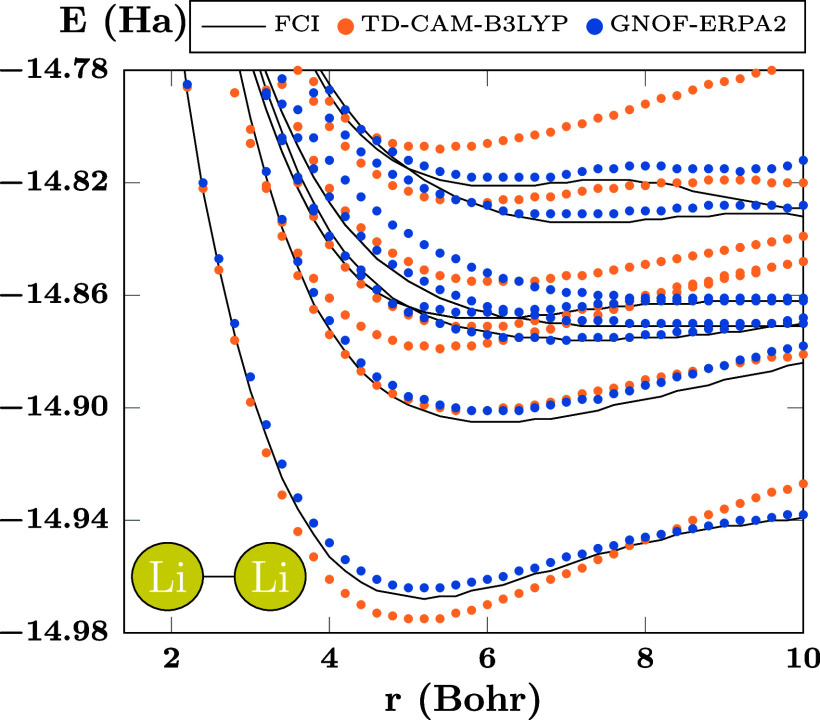
PECs of the first states of Li_2_ computed using FCI, TD-CAM-B3LYP, and GNOF-ERPA2. There are *N*_cwo_ = 10 orbitals paired to each strongly double-occupied orbital.

Finally, Figure 9 presents a comparison of the results of GNOF-ERPA2 (blue circles) with those of restricted TD-CAM-B3LYP (orange circles) and FCI (black lines). It can be seen that TD-CAM-B3LYP recovers a certain resemblance to the profile of the FCI curves, but there is a significant quantitative difference in favor of GNOF-ERPA2. Moreover, TD-CAM-B3LYP PECs show an overestimation of the electron correlation in the bonding region, but the PECs cross the lines of FCI as the correlation is underestimated in the dissociation process. This can be improved but not completely corrected by an unrestricted TD-CAM-B3LYP calculation, as shown in the Supporting Information, at the price of spin contamination. A great advantage of GNOF is that its PECs tend to be parallel to the FCI PECs as a consequence of the balanced treatment of electron correlation, without introducing spin contamination.

**Figure 10:**
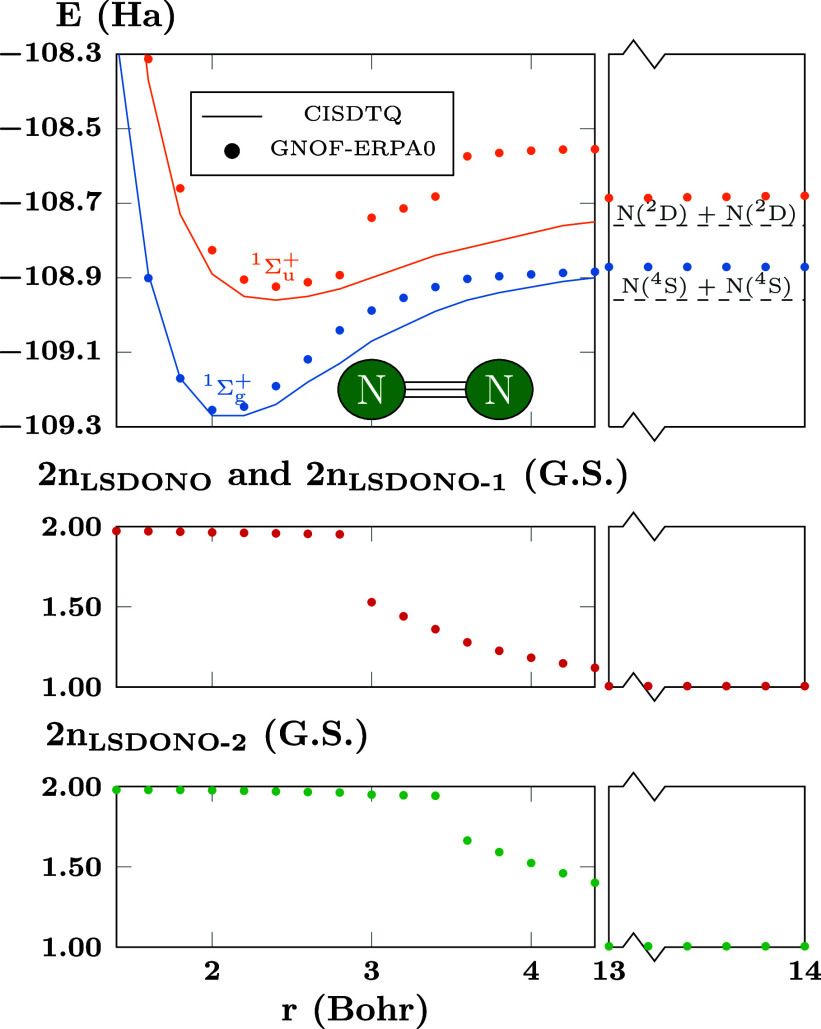
PECs of N_2_ computed using GNOF-ERPA0 and CISDTQ. There are *N*_cwo_ = 3 orbitals paired to each strongly double-occupied natural orbital. The top panel presents the energy of the ground and the first-excited state. The dotted lines corresponds to the FCI energy of two N(^4^S) atoms and two N(^2^D) atoms. The middle panel corresponds to the occupation numbers of the LSDONO and LSDONO-1, and the bottom panel corresponds to the occupation numbers of the LSDONO-2 for the ground state.

### 4.3 Multiple Bonds: N_2_

The molecular nitrogen provides a challenging system, as a triple bond is involved, allowing testing of the capabilities and limitations of the current PNOF-ERPA implementation. The PECs of the ground state ^1^Σ_g_^+^ and of the first excited state ^1^Σ_u_^+^ were calculated using a cc-pVDZ basis set and are presented in the top panel of Figure 10, with the solid lines corresponding to a CISDTQ calculation that is very close to the reported values of FCI in the bonding region,^65^ and the circle marks corresponding to the GNOF-ERPA0 results. The FCI values of the ground and first excited states at the dissociation limit are indicated in dashed lines.

In order to analyze the results, it is convenient to divide the dissociation in three zones, the first corresponding to a separation distance below 2.8 Bohr and containing the bonding region, characterized by occupation numbers close to “two” for the strongly double-occupied natural orbitals. The second region corresponds to the interval between 2.8 and 3.6 Bohr, and is characterized by the lowest strongly double-occupied natural orbitals (LSDONO) becoming fractional occupied as can be seen in the red curve at the middle panel. A similar behavior is obtained for the LSDONO-1, which together with LSDONO represents the bond breaking process of the two π orbitals. The third region appears for distances beyond 3.6 Bohr, and is characterized by the occupation numbers of the LSDONO-2 becoming fractional, as can be seen in the green curve at the bottom panel of Figure 10. This time, the process corresponds to breaking of the σ orbital. Finally, as the separation distance of the nitrogen atoms increases, the occupation numbers of LSDONO, LSDONO-1 and LSDONO-2 move to values close to “one”, which together with the coupled weakly occupied natural orbitals represents the complete dissociation to two N(^4^S) atoms.

Regarding the first region, GNOF achieves remarkable success by providing by itself energies that are very close to the CI results for both the ground state and the first excited state. For the second and third regions, the ground state predicted by GNOF remains close to the CI results, although the change of the occupation numbers at 2.8 Bohr for LSDONO and LSDONO-1, and at 3.6 Bohr for LSDONO-2 is not smooth. This behavior of GNOF is already known when moving from electron correlation regime,^50^ and is reflected in the first excited state that presents nonsmooth transitions exactly in these values of the separation distance.

Finally, it is important to mention that achieving the correct excitation energies in the second and particularly in the third region is difficult due to the fact that there are occupation numbers with the same value, for example, those of the LSDONO and the LSDONO-1. This is particularly significant at the dissociation limit, where there are six occupation numbers with almost the same value of “one”; therefore, the **ΔN** matrix presents several zeros and becomes noninvertible. On the right side of the plot, we present selected points of the first excited state. GNOF provides an excitation energy of 0.19 Ha in good agreement with the value of 0.20 Ha provided by FCI. However, we still want to highlight that the current algorithm becomes unstable in this scenario. We attribute these difficulties not to inaccuracies in the GNOF-ERPA approach, but to the fact that the Δ*N* matrix may be ill-conditioned and that several algebraic techniques should be explored for these cases.

## 5 Conclusions

This work validates the coupling of PNOF functionals with the ERPA equations as a very promising approach for studying excited states. As expected, the switch from ERPA0 to ERPA1 and ERPA2 improves the results. It is important to note that ERPA0 has shown inaccuracies regarding avoided crossings in the studied systems, and although ERPA1 improves the results, ERPA2 has been required to describe them correctly. Despite this fact, ERPA0 has been able to correctly describe crossings between curves and provides a general depiction of the excited states.

Regarding the functionals tested, PNOF5 seems to be enough for small molecules, but as the size of the systems increases, the interpair electron correlation becomes important, and PNOF7 and GNOF provide better results. The PNOF-ERPA approach becomes promising in the context of the other methods used for excited states, as PNOF provides values comparable to those of high-levels of CI. We must recall that the cost of a ground state PNOF calculation is of the fourth order with the number of orbitals for the ground state, while the cost of calculating the CI wave function depends on the number of determinants with exponential growth. In fact, the scaling of the ground-state PNOF calculation is comparable with that of hybrid density functional approximations, with the advantage of the PNOF being able to deal simultaneously with charge delocalization and static correlation. Once the ground-state PNOF result has been achieved, the scaling of the excited-state calculation becomes of the sixth order, comparable to that of standard TD-DFT, but with substantially better results, as shown in this work.

The capabilities of PNOF calculations have now been extended to all chemical problems that involve excited states; for example, in the future, the study of photochemical processes may benefit from a balanced inclusion of static and dynamic correlation. It is expected that the accuracy of the excited states will be greatly benefited by the development of better functionals that surpass the currently good performance of the GNOF. Finally, as the potential of the PNOF-ERPA approach has been established, it is desirable to develop the implementation that avoids the diagonalization of the full matrix, as well as taking care of challenging cases with degeneracy on the values of the occupation numbers.

## Supporting Information Available

Unrestricted TD-CAM-B3LYP calculations of Li_2_ and N_2_ molecules (PDF)
