# Supplementary material for: Excited States by Coupling Piris Natural Orbital Functionals with the Extended Random-Phase Approximation
Source: J Chem Theory Comput. 2024 Feb 14;20(5):2140–51. doi: 10.1021/acs.jctc.3c01194 (PMC10938499; doi:10.1021/acs.jctc.3c01194)
Supplement: Supplementary file 1 — ct3c01194_si_001.pdf [file ct3c01194_si_001.pdf]

Supporting Information: Excited states by coupling Piris natural orbital functionals with the extended random phase approximation

Juan Felipe Huan Lew-Yee,[*,†,‡] Iván Alejandro Bonfil-Rivera,[†] Mario Piris,[*,‡,¶,§] and Jorge M. del Campo[*,†]

†*Departamento de Física y Química Teórica, Facultad de Química, Universidad Nacional Autónoma de México, México City, C.P. 04510, México*

‡*Donostia International Physics Center (DIPC), 20018 Donostia, Spain.*

¶*Kimika Fakultatea, Euskal Herriko Unibertsitatea (UPV/EHU), 20018 Donostia, Spain.*

§*IKERBASQUE, Basque Foundation for Science, 48013 Bilbao, Spain.*

E-mail: felipe.lew.yee@quimica.unam.mx; mario.piris@ehu.eus; jmdelc@unam.mx

Abstract

In this Supporting Information we present the unrestricted TD-DFT calculations of Li_2 and N_2, for comparison with the PNOF-ERPA results provided in the main text. Despite that unrestricted TD-DFT calculations may provide better potential energy curves compared to the restricted TD-DFT calculations, it comes at the price of spin contamination. A great advantage of GNOF is that its PECs tend to be parallel to the Full-CI PECs as a consequence of the balanced treatment of electron correlation, while at the same time avoids the spin contamination.

Unrestricted TD-DFT Calculations

Results of calculations performed with an unrestricted TD-CAM-B3LYP calculation.

Li_2

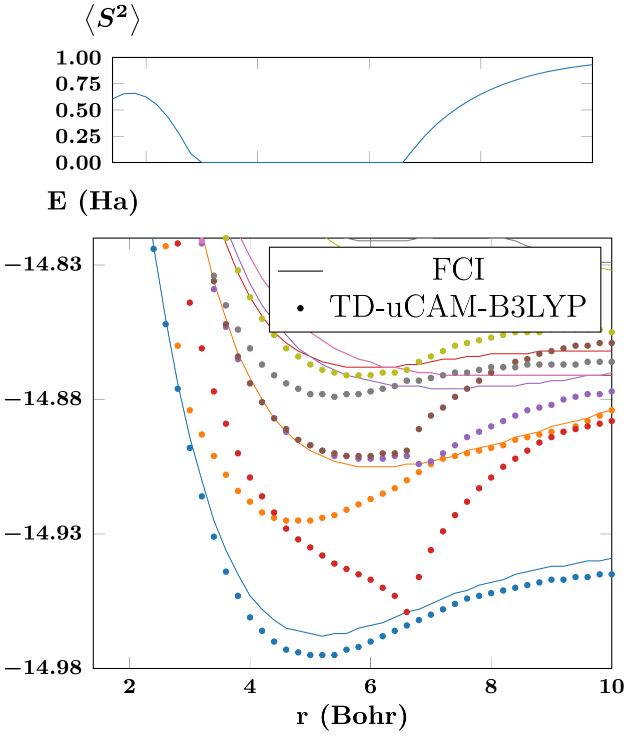

Figure S1: Top panel: Values of $\langle S^2 \rangle$ along the PEC. Bottom Panel: PECs of the first states of Li_2 computed using FCI (solid lines) and unrestricted TD-CAM-B3LYP (circle marks).

N_2

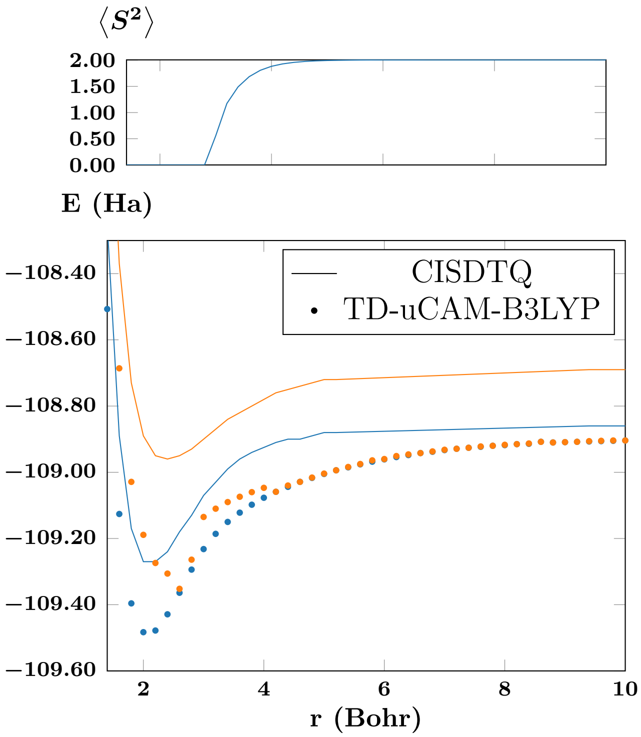

Figure S2: Top panel: Values of $\langle S^2 \rangle$ along the PEC. Bottom Panel: PECs of the first states of N_2 computed using CISDTQ (solid lines) and unrestricted TD-CAM-B3LYP (circle marks) with the cc-pVDZ basis set.
